# Type IV pilus retraction is required for *Neisseria musculi* colonization and persistence in a natural mouse model of infection

**DOI:** 10.1128/mbio.02792-23

**Published:** 2023-12-12

**Authors:** Katherine A. Rhodes, María A. Rendón, Man Cheong Ma, Al Agellon, Andrew C.E. Johnson, Magdalene So

**Affiliations:** 1 Immunobiology Department, University of Arizona College of Medicine, Tucson, Arizona, USA; 2 BIO5 Institute, University of Arizona, Tucson, Arizona, USA; 3 School of Animal and Comparative Biomedical Sciences, University of Arizona, Tucson, Arizona, USA; Max-Planck-Institut fur terrestrische Mikrobiologie, Marburg, Germany

**Keywords:** Type IV pilus retraction, PilT, PilU, PilT2, *Neisseria musculi*, *Neisseria gonorrhoeae*, *Neisseria meningitidis*, commensal *Neisseria*, colonization, persistence

## Abstract

Retraction of the pathogenic *Neisseria* Type IV pilus (Tfp) requires PilT, the primary retraction motor, and its paralogs PilU and PilT2. The importance of Tfp retraction for natural infection is unknown. Using our natural animal model of *Neisseria*-host interaction, we have examined the role of these proteins in the ability of commensal *Neisseria musculi* (Nmus) to colonize and persist in its native host, the mouse. We report that Nmus Δ*pilT* cannot colonize mice; Δ*pilU* and Δ*pilT2* can colonize and persist in mice, but in lower numbers. Microcolonies formed by Δ*pilT*, Δ*pilTU*, and *pilT_L201C_*, expressing PilT with a point mutation in its ATP hydrolysis domain, are sensitive to removal by fluid shear forces. Thus, PilT promotes Nmus colonization while PilU and PilT2 influence persistence. We present a non-exclusive model for how these Tfp retraction motor proteins contribute to Nmus persistent colonization. Our findings have implications for the roles of these motor proteins in mediating interactions of human-adapted pathogenic and commensal *Neisseria* with their human host.

**IMPORTANCE:**

We describe the importance of Type IV pilus retraction to colonization and persistence by a mouse commensal *Neisseria, N. musculi,* in its native host. Our findings have implications for the role of Tfp retraction in mediating interactions of human-adapted pathogenic and commensal *Neisseria* with their human host due to the relatedness of these species.

## INTRODUCTION

The Type IV pilus (Tfp) is a multifunctional structure that spans the cell envelope of many Gram-negative bacteria (1). Tfp enables DNA uptake, adherence, surface motility, and interbacterial and bacteria-host cell signaling. These processes are vital for bacterial survival in a variety of environments.

Tfp contributes to the virulence of human pathogens *Neisseria meningitidis* (Nme) and *Neisseria gonorrhoeae* (Ngo) (2–7). The pilus fiber, consisting of the major subunit pilin (PilE) and minor pilins (8–10), promotes initial contact of Ngo and Nme with human epithelial cells (11–14). Forces generated by cycles of pilus retraction and extension trigger mechanosensitive host pathways that modulate responses to infection (15–18). In Nme, Tfp promotes efficient colonization of the endothelium (19). In an engrafted mouse sepsis model (20), Tfp retraction promotes bacterial release from the endothelium, contributing to bacteremia. In Ngo, Tfp-dependent surface motility drives microcolony and biofilm formation (21).

Tfp fiber extension requires PilF, while retraction relies on PilT and its paralogs PilU and PilT2 (8, 22). These retraction motor homologs belong to the AAA+ ATPase family of proteins that form homohexameric rings (23, 24) that interact with the inner membrane Tfp platform complex (1, 25–27). ATP hydrolysis by PilT family proteins is proposed to rotate the Tfp platform protein PilC (PilG in *Neisseria*), followed by disassembly of pilins and their repositioning into the membrane (9, 28–31).

Ngo Δ*pilT* is non-motile, nearly non-transformable, and incapable of activating mechanosensitive host pathways (17, 18, 32). While PilT-independent retraction can occur from collapse of the pilus fiber into the secretin pore, these events generate less force than retraction powered by PilT (33, 34). Modifying ATP hydrolysis by PilT changes the ability of Ngo to manipulate its environment (35). A leucine to cysteine substitution in PilT Walker B (PilT_L201C_) reduces the ATP hydrolysis rate of this enzyme by 50% and the ability of the mutant to form microcolonies and activate host signaling pathways. Mutagenesis of PilU and PilT2 likewise demonstrates their role in determining retraction speed and bacterial motility, adhesion, and virulence (36–39). These findings mirror observations made in other bacteria: mutation of *pilT* and its paralogs in *Pseudomonas aeruginosa* and *Vibrio cholerae* alters bacterial behavior (24, 40, 41).

How PilT, PilU, and PilT2 influence Ngo and Nme interactions with the human host is unclear, as suitable animal models are lacking. Furthermore, their roles in commensal *Neisseria* biology are unknown. We recently developed a natural mouse model for *Neisseria* colonization and persistence using mouse commensal *Neisseria musculi* (Nmus) that allows us to examine the function of genes conserved between Nmus and other *Neisseria* species (42, 43). As PilT, PilU, and PilT2 are among these highly conserved genes, this model system allows us to assess the role of these motor proteins in *Neisseria*-host interactions.

## RESULTS

### PilT, PilU, and PilT2 of *N. musculi* and human-adapted *Neisseria* are highly related

**Fig 1:**
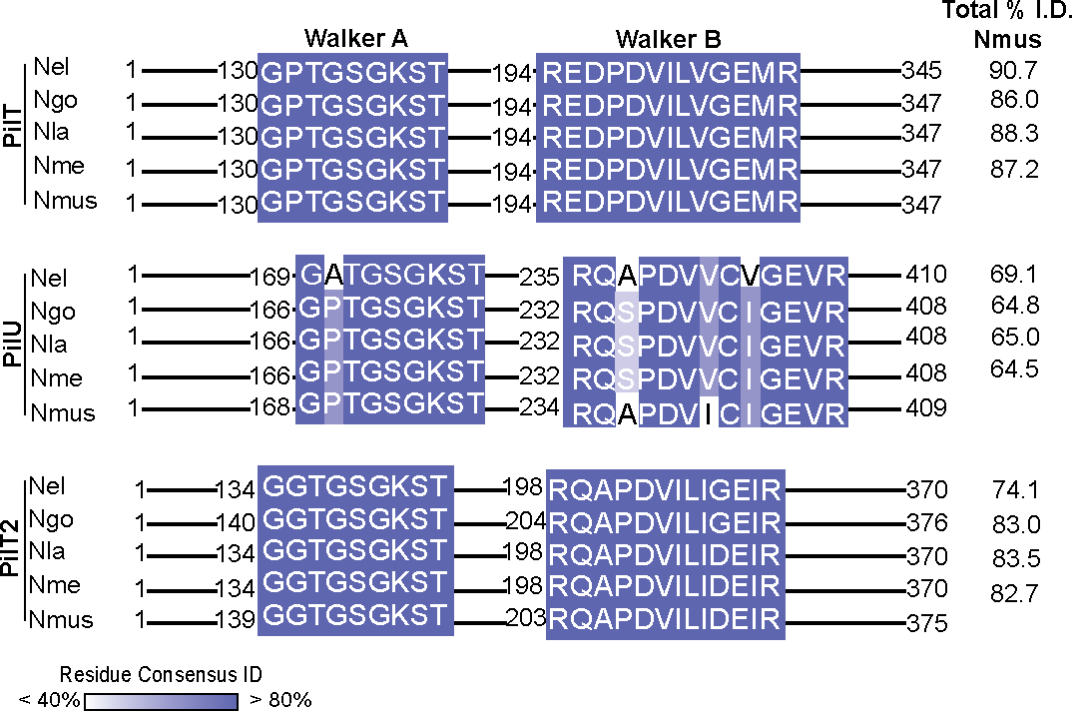
Walker A and B boxes of PilT, PilU, and PilT2 of *N. musculi* and human-adapted *Neisseria* species are highly conserved. Alignment of amino acid sequences in the Walker A and B boxes of Nmus PilT, PilU, and PilT2 with orthologs encoded by *N. lactamica* 002-06 (Nla)*; N. elongata glycolitica* ATCC 29315 (Nel)*; N. meningitidis* MC58 (Nme); and *N. gonorrhoeae* FA1090 (Ngo). The percent identity of each residue to the consensus sequence is shaded in blue. For each ortholog, the total percent identity to Nmus PilT, PilU, or PilT2 is listed on the right. Query coverage for all comparisons was 97–99%. A complete alignment of all amino acids for each ortholog can be found in Fig. S1.

BlastP and clustalW analyses show Nmus PilT, PilU, and PilT2 are highly homologous to orthologs in Ngo, Nme, and commensals *Neisseria elongata* (Nel) and *Neisseria lactamica* (Nla) (Fig. S1; Fig. 1). In all tested species, PilT and PilT2 have high amino acid similarity overall (74–90% identity to Nmus, 97–99% query coverage); their Walker A and B domains are identical, and each protein possesses putative Asp and His box domains indicative of the Type II ATPase family. Although Nmus PilU is less like its orthologs (64.5–69.1% identity, 99% query coverage), all PilU Walker A and B boxes and Asp and His motifs within the core NTPase domain vary only by 1–2 amino acids (23). The ATP binding motifs in these motor proteins are conserved in their homologs in other species, as indicated by sequence analysis (Fig. S1). In PilT, the highest sequence divergence was observed in the C-terminal NTPase domain. Within PilU, dissimilarity between Nmus and the human-adapted species was highest in the final 100 residues of the protein. Regarding PilT2, the highest dissimilarity was observed in the final 100 residues of the C-terminal domain (like PilU and PilT) and the N-terminal domain predicted to facilitate membrane interaction.

### *N. musculi* PilT, PilU, and PilT2 are important for different stages of infection

We created an in-frame deletion of Nmus *pilT* and measured its ability to colonize and persist in mice in our mouse model of *Neisseria* colonization and persistence (43–45). Nmus Wt, Δ*pilT*, and its complement were inoculated into the oral cavity of CAST/EiJ and A/J mice. Oral swabs and fecal pellets were plated weekly, over 8 weeks, to assess colonization and persistence. Colonization is defined as the presence of CFU for two consecutive weeks by week 4. Persistence is defined as the presence of wild-type CFU levels for more than three consecutive weeks by week 8.

**Fig 2:**
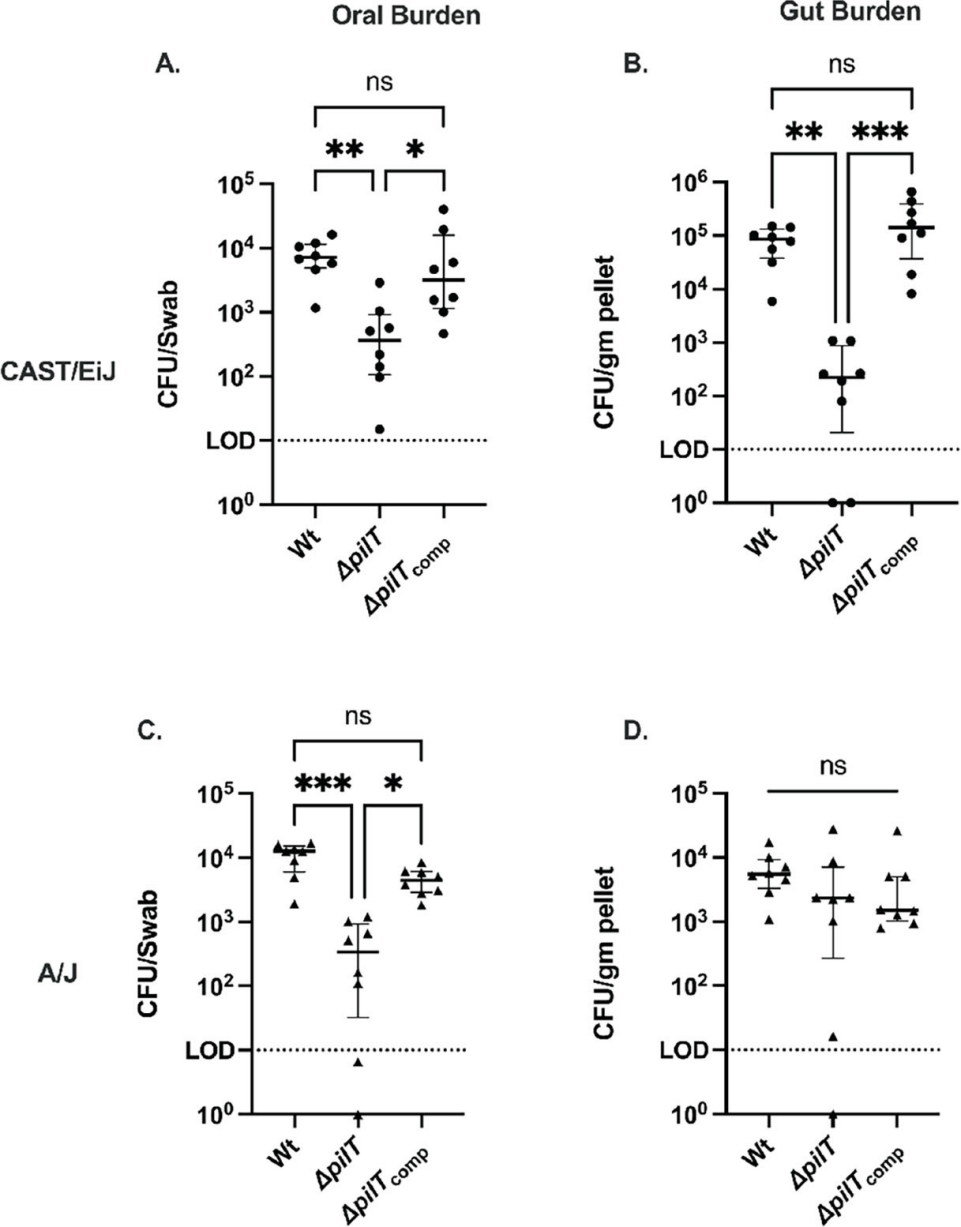
Loss of primary retraction motor PilT results in decreased *N. musculi* burdens in CAST/EiJ and A/J mice. Nmus Wt, Δ*pilT,* and complemented strains were inoculated into the oral cavity of CAST/EiJ and A/J mice, and oral swabs and fecal pellets were collected weekly for 8 weeks. Points represent average weekly Nmus burden per group, with lines denoting the interquartile range and median. (**A**) Oral burdens of Wt, Δ*pilT*, and Δ*pilT*_comp_ in CAST/EiJ mice. (**B**) Gut burdens of Wt, Δ*pilT*, and Δ*pilT*_comp_ in CAST/EiJ mice. (**C**) Oral burdens of Wt, Δ*pilT*, and Δ*pilT*_comp_ in A/J mice. (**D**) Gut burdens of Wt, Δ*pilT*, and Δ*pilT*_comp_ in A/J mice. Statistical comparison was conducted using Kruskal-Wallis ANOVA with Dunn’s post-test to compare overall group burdens to Wt controls. *N* = 18–20 mice per group. *P < 0.05, **P < 0.01, ***P < 0.001, ****P < 0.0001.

Δ*pilT* is impaired in colonizing CAST/EiJ and A/J mice compared to Wt and its complement (Table 1). Moreover, in the few Δ*pilT*-colonized CAST/EiJ mice, mutant burdens in the oral cavity and gut were significantly reduced (Fig. 2A and B). Burdens were similarly reduced in the oral cavity but not the gut of A/J mice (Fig. 2C and D). These differences in gut burden may reflect differences in bacterial behavior in the two host environments. In this context, it should be noted that Wt gut burdens were also lower in A/J than CAST/EiJ mice.

**TABLE 1:** Mutations to PilT, the primary pilus retraction motor, decrease *N. musculi* colonization frequency in CAST/EiJ and A/J mice^*a*^

Strain	Colonization frequency CAST/EiJ mice	Colonization frequency A/J mice
Wt	80% (*n* = 20)	88% (*n* = 18)
Δ*pilT*	20% (*n* = 20)****	32% (*n* = 19)***
Δ*pilT*_comp_	70% (*n* = 10) n.s.	70% (*n* = 10) n.s.

^*a*^ Colonization was determined at 4 weeks post-inoculation. Statistical analysis was conducted by pairwise Fisher’s exact test of Wt with *ΔpilT* and complemented strains. Group size is denoted in parenthesis. ****P < 0.0001, ***P < 0.001. n.s., not significant.

The colonization deficiency of Δ*pilT* led us to test Δ*pilU,* Δ*pilTU*, and Δ*pilT2* for their behavior *in vivo.* We focused on the oral cavity in these studies because this site in humans is inhabited by commensal *Neisseria*. We also focused on A/J mice because the Δ*pilT* phenotypes in A/J and CAST mice are very similar.

**Fig 3:**
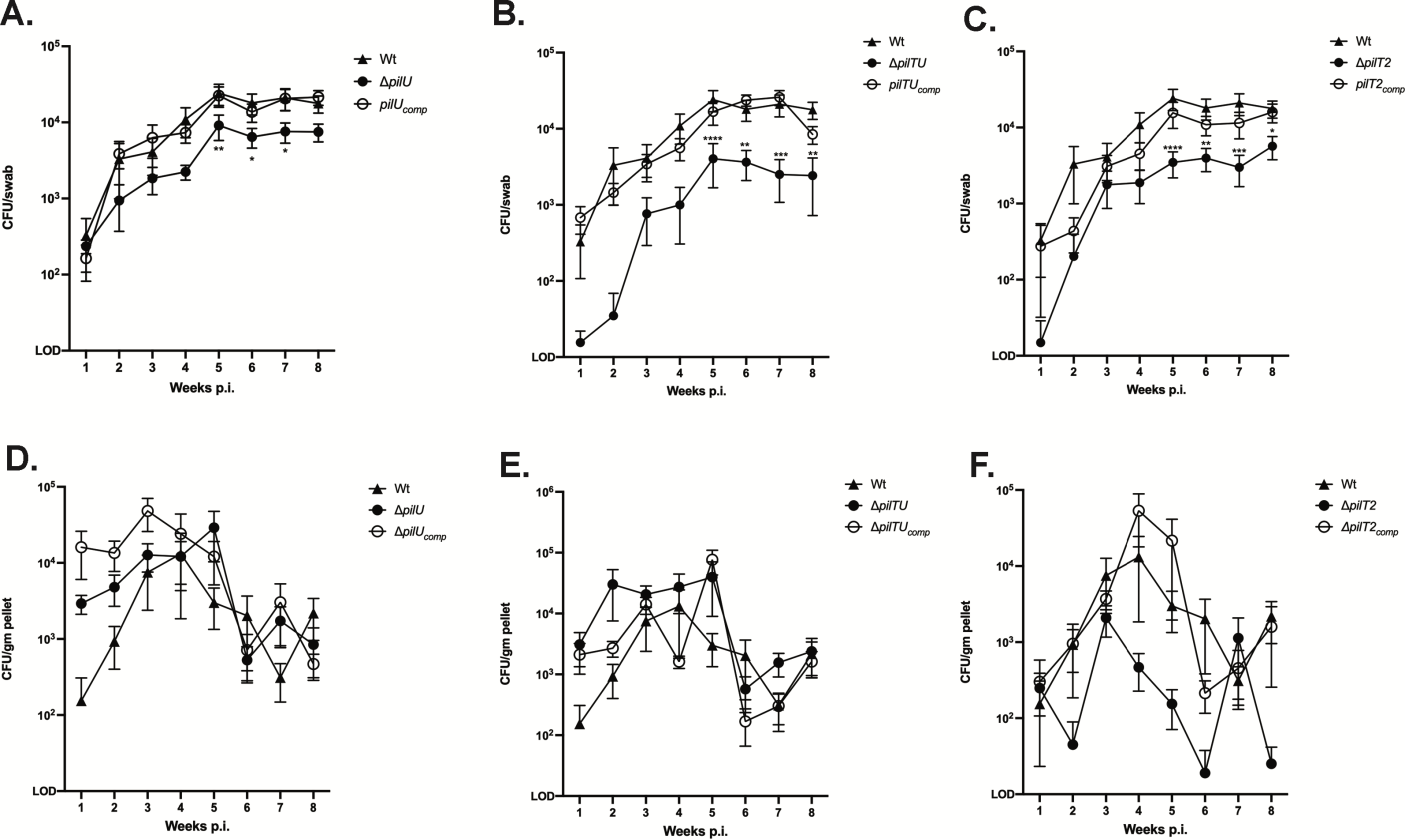
Deletion of *pilT* paralogs reduces the number of *N. musculi* colonizing the A/J mouse oral cavity but not the gut. Nmus Wt, Δ*pilT2,* Δ*pilU,* Δ*pilTU*, and complemented strains were inoculated into the oral cavity of A/J mice, and oral swabs (**A–C**) and fecal pellets (**D–F**) were collected weekly for 8 weeks. Data are presented as mean group burden at each time point, ±SEM. *N* = 10 mice per group. *P < 0.05, **P < 0.01, ***P < 0.001, ****P < 0.0001. Two-way ANOVA with Dunnett’s post-test was used to examine differences in mean weekly burden between mutant and Wt controls. No significant differences were observed in gut samples.

Unlike Δ*pilT*, Δ*pilU* colonized mice like Wt (Table 2), while Δ*pilTU* and Δ*pilT2* colonized at moderately but not significantly reduced frequencies. Beginning 4 weeks post-inoculation (p.i.), fewer CFUs were recovered from mice for all three mutants, with Δ*pilTU* CFUs being the most reduced (Fig. 3A through C). There were no significant differences in their gut burdens (Fig. 3D through F). These results strongly suggest that PilT is essential for colonization, while PilU and PilT2 influence persistence.

**TABLE 2:** Mutations to PilT paralog motors do not significantly impact colonization frequency of the oral cavity in A/J mice^*a*^

Strain	Colonization frequency A/J mice
Wt	80% (*n* = 10)
Δ*pilU*	100% (*n* = 10) n.s.
Δ*pilU*_comp_	90% (*n* = 10) n.s.
Δ*pilT2*	60% (*n* = 10) n.s.
Δ*pilT2*_comp_	90% (*n* = 10) n.s.
Δ*pilTU*	60% (*n* = 10) n.s.
Δ*pilTU*_comp_	100% (*n* = 10) n.s.

^*a*^ Colonization was determined at 4 weeks post-inoculation. Statistical analysis was conducted by pairwise Fisher’s exact test to compare Wt with Δ*pilT2*, Δ*pilU*, and Δ*pilTU* and complemented strains. Group size is denoted in parentheses. n.s., not significant.

### Stable colonization requires a fully functional PilT

We reported that Ngo PilT_L201C_, which harbors a *pilU-*like Walker Box B, displays a reduced ability to hydrolyze ATP and interacts aberrantly with host cells *in vitro* (35). To determine the impact an analogous mutation in Nmus has *in vivo,* we created Nmus *pilT_L201C_* and measured oral cavity and gut colonization of two independently derived mutants, *pilT_L201C_* 1813 and *pilT_L201C_* 1824, in CAST/EiJ mice (Fig. S2). Both mutants colonized mice like Wt, and their oral cavity and gut burdens were similar to Wt controls (Fig. S2A through C).

Curiously, in 55–68% of CAST/EiJ mice, oral cavity and gut burdens of the *pilT_L201C_* mutants fluctuated throughout the study (Fig. S2D). Increases in CFUs were followed by >1.5 log decreases in burden in consecutive cycles (Fig. S2E through G). Bacterial population size was highly variable in the Walker Box B mutant: the mean standard deviation of bacterial counts across the entire study was significantly higher in *pilT_L201C_* -inoculated mice compared to Wt (*P* < 0.01) (Fig. S2H and I). This phenotype was observed in 100% of Δ*pilT*-colonized mice but in only 30% of Wt-colonized mice (Fig. S2J). We have named this behavior the cyclic detection phenotype. These results strongly suggest that the PilT Walker Box B domain is important for stable, long-term colonization.

The endogenous gut microbiota prevents colonization by exogenous intruders and controls outgrowth of pathobiont populations through competitive and host-modulatory mechanisms (46). To determine if microbiota composition was associated with colonization resistance or variability, we analyzed the alimentary tract microbiota of a subset of inoculated mice. No difference in community composition was found between stably and cyclically colonized mice or between colonized and uncolonized mice (Fig. S3A through D). Therefore, the microbiota appears to have had little influence on colonization.

Finally, to exclude the possibility that the colonization phenotypes displayed by retraction mutants were caused by decreased fitness, we compared the growth kinetics of our panel of mutants to Wt and complemented controls. No significant difference in their growth rate was observed (Fig. S4A).

### Δ*pilT* cannot retract pili and is hyperpiliated

We determined whether our Nmus mutants are capable of retracting Tfp, using DNA uptake as a readout (43). Compared to controls, transformation frequencies of Δ*pilT* and Δ*pilTU* were significantly decreased, and Δ*pilU*, *pilT*_*L201C*_, and Δ*pilT2* were slightly lower (Table 3). This strongly suggests that Δ*pilT* cannot retract Tfp, while the other mutants have retained this ability, at least partially.

**TABLE 3:** Transformation frequency of *N. musculi* pilus retraction mutants and complemented controls^*a*^

Strain	Transformation frequency ± SD	Transformation frequency (% Wt)
Wt	7.74 × 10^−3^ ± 3.87 x 10^−3^	–
Δ*pilT*	<8.44 x 10^−8*a*^, n.d.	<0.01
Δ*pilT*_comp_	5.40 × 10^−3^ ± 4.11 x 10^−3^	70
*pilT_L201C_*1813	5.21 × 10^−3^ ± 5.43 x 10^−3^	67
*pilT_L201C_*1824	8.30 × 10^−3^ ± 5.62 x 10^−3^	67
Δ*pilU*	4.95 × 10^−3^ ± 2.79 x 10^−3^	64
Δ*pilU*_comp_	9.92 × 10^−3^ ± 7.50 x 10^−3^	128
Δ*pilT2*	5.72 × 10^−3^ ± 5.76 x 10^−3^	74
Δ*pilT2*_comp_	5.41 × 10^−3^ ± 2.76 x 10^−3^	70
Δ*pilTU*	<8.44 x 10^−8*a*^, n.d.	<0.01
Δ*pilTU*_comp_	6.91 × 10^−3^ ± 1.22 x 10^−3^	89

^a^ *P <* 0.05, one-way ANOVA with Dunnet’s multiple comparison test to compare mean transformation frequency to Wt controls. The limit of detection is 8.44 x 10^−8^; all tests were conducted in four biological replicates. n.d., not determined.

Ngo Δ*pilT* overexpresses *pilE* and overproduces pilin and pilus fibers (35). We examined our retraction mutants for *pilE* mRNA and pilin levels in semi-purified pili preparations (Fig. S4B and C). Nmus Δ*pilT* produced significantly more *pilE* transcript and pili than controls, while *pilT_L201C_* and Δ*pilU* produced normal levels of *pilE* transcript and pili. Δ*pilTU* and Δ*pilT2 pilE* mRNA levels were moderately but not significantly increased. Scanning electron microscopy showed an increased number of pilus-like fibers on the outer surface of Δ*pilT* cells compared to Wt controls (Fig. S4D). Thus, loss of PilT in Nmus also leads to hyperpiliation (35).

### Δ*pilT* and Δ*pilTU* are deficient in adhering to mouse epithelial cells

**Fig 4:**
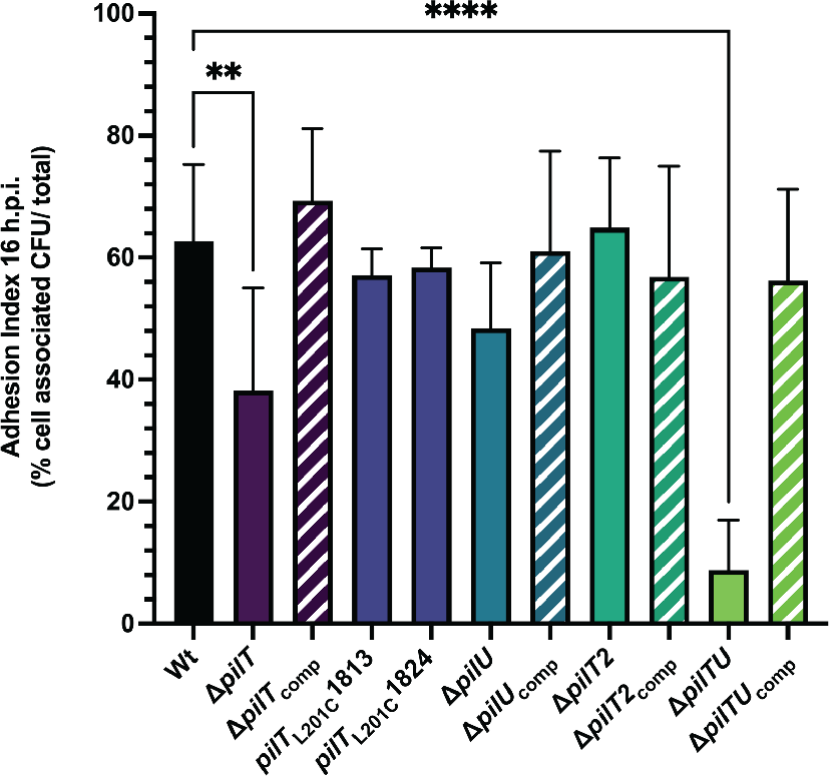
Tfp retraction affects *N. musculi* adhesion to mouse epithelial cells. Adhesion of Nmus Wt and retraction motor mutants to CMT-93 mouse epithelial monolayers, 16 hours post-inoculation. Bars represent mean adhesion index ±SD of 4–8 independent biological replicates conducted in technical triplicate. **P < 0.01, ****P < 0.0001, ordinary one-way ANOVA with Dunnett’s comparison to Wt controls.

We determined whether the colonization phenotypes of the retraction mutants are reflected in host cell adherence. CMT-93 cells were infected with our panel of mutants, and their adhesion indices [(# cell-associated CFUs/# inoculated CFUs) × 100] were determined at 4 and 16 hours p.i. At 4 hours, all strains adhered variably; although adhesion trended lower than controls, the differences were not statistically significant (Fig. S4E). At 16 hours, significantly fewer Δ*pilT* and Δ*pilTU* adhered to cells compared to controls, while the other mutants adhered as well as controls (Fig. 4). Consistent with the mouse colonization phenotypes (Tables 1 and 2), long-term adherence to CMT-93 cells requires full activity of the PilT motor.

### Retraction mutants are defective in microcolony formation

**Fig 5:**
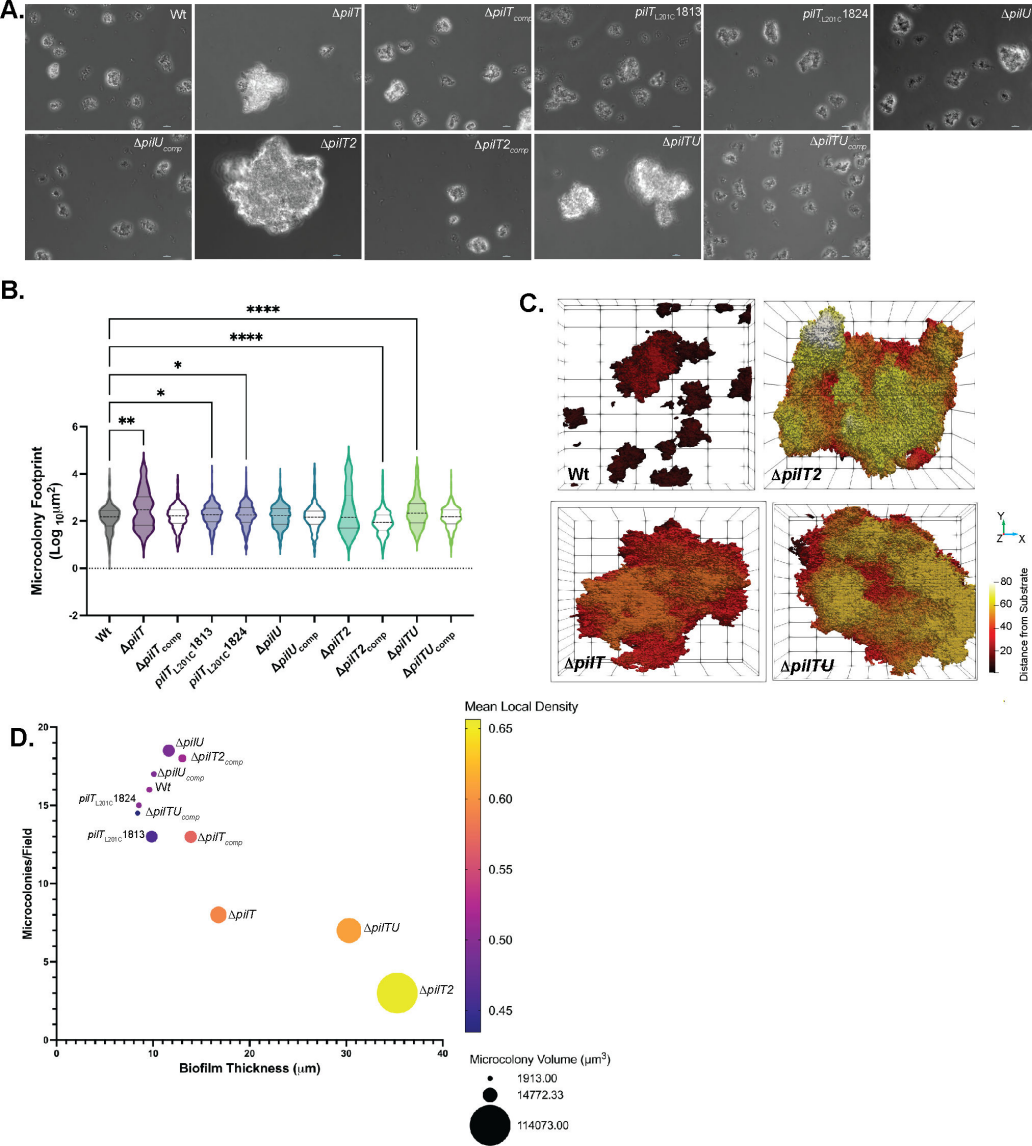
*N. musculi* microcolony structure is altered by changes to Tfp retraction. (**A**) Representative phase contrast micrographs at 400X of Nmus strains, 24 hours post-inoculation. Scale bar: 10 µm. (**B**) Area of microcolony footprint of Nmus strains after 24 hours of growth in Ibidi channel slides. Violin plots represent the distribution of all measured microcolony areas captured from 7 to 8 representative fields per replicate from three independent biological replicates. Lines indicate the median and interquartile range. Statistical analysis was conducted by Kruskal-Wallis test with Dunn’s comparison to Wt controls. (**C and D**) Slides shown in (**A**) were stained with DAPI and imaged on a Zeiss 880 Laser scanning confocal microscope at 630×. (**C**) Z stacks from 12 to 14 fields per strain were analyzed using BiofilmQ, and the results were rendered using Paraview. Images shown are representative renderings from Wt, Δ*pilT*, Δ*pilT2*, and Δ*pilTU* isolates. Distance of image from the slide surface is color-coded from dark red to yellow. (**D**) Plot summarizing BiofilmQ results. Microcolony volume (μm^3^) is represented by dot size, and average local cell density is represented by color. Cell density measurements were calculated as cells/μm^3^ in a 50-voxel sphere.

As microcolony formation follows initial *Neisseria* contact with host cells (47, 48), we measured microcolony formation by our mutants. Cells grown in channel slides under static conditions for 24 hours were examined by phase contrast microscopy. All mutant microcolonies were noticeably larger than control microcolonies (Fig. 5A). Reflecting this size difference, all except Δ*pilU* microcolonies have a larger median area at the slide surface (Fig. 5B).

We next examined the three-dimensional structures of mutant microcolonies by confocal microscopy. Δ*pilT*, Δ*pilTU*, and Δ*pilT2* formed fewer microcolonies than controls (17 vs 5–8 microcolonies per field; *P* < 0.01–0.001, one-way ANOVA with Dunnett’s comparison to controls) (Fig. S5A). Mutant structures have larger volume and higher cell densities (Fig. 5C; Fig. S5B through E). Figure 5D summarizes the phenotypes of microcolonies formed by our panel of mutants. Δ*pilT*, Δ*pilTU*, and Δ*pilT2* stood apart in all parameters examined, forming structures that are fundamentally different from Wt.

### Δ*pilT* and Δ*pilTU* microcolonies are sensitive to removal by fluid shear stress

While assessing microcolony biomass by crystal violet (CV) retention, we noticed differences in CV staining of mutant microcolonies (Fig. S5F). We suspected that these microcolonies may be adhering poorly to the microtiter plates and detaching during sample processing. To test this, we seeded our mutants on channel slides and enumerated CFUs removed from the slides upon exposure to fluid flow at rates producing 0.2 and 0.6 dynes of shear force, rates used in a study of Nme adhesion (49). Relative to controls, significantly more Δ*pilT*, *pilT*_*L201C*_ 1813, and Δ*pilTU* detached from the slide at flow producing 0.6 dynes/cm^2^; Δ*pilTU* also detached at 0.2 dynes/cm^2^ (Table 4). The other mutants had a slight but not statistically significant tendency to detach from the substrate at 0.6 dynes/cm^2^ of force. Thus, microcolonies formed by cells without PilT or cells expressing PilT_L201C_ formed fragile attachments to the substrate and were more susceptible to removal by fluid shear stress. Moreover, the hyperpiliated state of Δ*pilT* cells could not compensate for this sensitivity to flow. These findings contrast with the behavior of Nme Δ*pilT*, which is more adherent under high shear stress (20, 47).

**TABLE 4:** Resistance to shear stress by Tfp retraction motor mutants^*a*^

Strain	Mean detached CFU 0.2 dynes/cm^2^	% Wt	*P* value	Mean detached CFU 0.6 dynes/cm^2^	% Wt	*P* value
Wt	4.65 ± 0.58	–	–	3.46 ± 0.53	–	–
Δ*pilT*	5.77 ± 1.32	124	0.19	5.17 ± 0.70	**150**	**0.01**
Δ*pilT*_comp_	4.99 ± 0.86	107	0.99	3.42 ± 0.36	99	0.99
*pilT*_L201C_1813	5.45 ± 0.30	117	0.46	5.14 ± 0.44	**149**	**0.01**
*pilT*_L201C_1824	6.01 ± 0.74	129	0.07	4.28 ± 0.98	124	0.44
Δ*pilU*	5.78 ± 0.21	124	0.13	4.13 ± 0.83	119	0.67
Δ*pilU*_comp_	4.81 ± 0.79	103	0.99	3.69 ± 0.71	107	0.99
Δ*pilT2*	5.20 ± 0.40	112	0.84	4.17 ± 0.58	121	0.60
Δ*pilT2*_comp_	4.61 ± 0.65	99	0.99	4.01 ± 0.76	116	0.84
Δ*pilTU*	6.16 ± 0.22	**133**	**0.03**	4.90 ± 0.88	**142**	**0.03**
Δ*pilTU*_comp_	5.65 ± 0.23	122	0.23	4.34 ± 0.72	125	0.36

^*a*^ Cultures incubated in Ibidi channel slides for 24 hours at static conditions were subjected to flow-producing force equivalent to 0.2 and 0.6 dyn/cm^2^, and effluent was plated to enumerate CFUs. Data represent average log_10_ CFU counts from four independent experiments, ±SD. Statistical analysis was conducted by two-way ANOVA of CFU counts with Dunnett’s comparison to Wt controls.

## DISCUSSION

In Ngo and Nme, PilT, PilU, and PilT2 are involved in Tfp retraction. Of these, only PilT has been examined in depth for its role in bacteria-host interactions. PilT is linked to Nme colonization of the vasculature and sepsis and promotes pro-survival responses in Ngo-infected epithelial cells (17, 19, 20, 35, 50). The importance of PilT, PilU, and PilT2 in commensal *Neisseria* infection biology is unknown despite the presence of these bacteria in the microbiota (51–53). Using *in vivo* and *in vitro* approaches, we have examined the contribution of the three retraction proteins to Nmus niche establishment in the mouse, its natural host (Table 5).

**TABLE 5:** Summary of pilus retraction motor mutant phenotypes^*a*^

Strain	Colonization and persistence	Burden	Pilin/*pilE* expression	Transformation frequency	16 hours adhesion	M.C. density	Surface detachment
Δ*pilT*	**⇓,λ**	**⇓**	**⇑**	**⇓**	**⇓**	**↑**	**⇑**
*pilT*_L201C_1813	**λ**						**⇑**
*pilT*_L201C_1824	**λ**						**↑**
Δ*pilU*		**⇓**		**↑**			**↑**
Δ*pilT2*	**↓**	**⇓**	**↑**			**⇑**	
Δ*pilTU*	**↓**	**⇓**	**↑**	**⇓**	**⇓**	**⇑**	**⇑**

^*a*^ All comparisons are made to Wt controls. Symbol definitions are as follows: ⇓, significant reduction; ⇑, significant increase; ↑, increase approaching significance; ↓, decrease approaching significance; λ, cyclic detection phenotype in CAST/EiJ mice. Empty cells indicate no change from Wt. M.C., microcolony.

PilT is necessary for Nmus colonization. Δ*pilT* poorly colonized the oral cavity of CAST/EiJ and A/J mice, compared to Wt and complemented controls. This defect is mirrored in their reduced ability to adhere to mouse cells. In the few mice harboring Δ*pilT*, mutant burdens were lower, and CFUs fluctuated over time. PilT-dependent Tfp retraction is therefore required for appropriate development of the Nmus community in the host environment. This idea is supported by the observation that *pilT_L201C_*, expressing a PilT with a point mutation in an ATP hydrolysis domain (Walker Box B), also has a cyclic detection phenotype. Ngo PilT_L201C_ hydrolyzes ATP at 50% of the Wt rate, and its activation of mechanosensitive host pathways is reduced. It is possible that the cyclic detection phenotype observed in Nmus *pilT*_L201C_ may arise in part through a similar mechanism. Future work will explore the connection between host pathway activation and PilT structure/function in commensal *Neisseria.*

The population size of Wt Nmus in C57Bl/6 mice was recently reported to fluctuate (54). The reason for this discrepancy in Nmus *in vivo* behavior in the two studies is unclear. It may be due to the different Nmus colony morphotypes used in the studies and/or differences in the microenvironments of the mouse strains. We demonstrated that the CAST/EiJ microbiota did not influence Nmus colonization; the first study did not examine this possibility. Differences in mouse immune responses may also influence colonization; such differences could account for our observation that Δ*pilT* colonizes the gut of CAST/EiJ and A/J mice differently. Pilin phase variation in Nmus, if it occurs, may account for some differences in colonization stability between the two studies and explain variation in adherence phenotypes that we observed between the two *pilT*_*L201C*_ mutants. It must also be noted that while piliation of Ngo *pilT*_*L201C*_ was moderately increased, piliation of Nmus *pilT_L201C_* mutants was unaffected (Nmus vs Ngo/Nme comparison; Table S1). More work is needed to address the influence of host environments on Nmus niche development and determine whether pilin phase variation occurs in Nmus.

Like Ngo (35), Nmus Δ*pilT* is hyperpiliated. Hyperpiliation of Ngo Δ*pilT* is thought to be responsible for increased adherence. In Nmus Δ*pilT*, however, hyperpiliation is inversely correlated with colonization and adherence, indicating that Tfp overproduction cannot compensate for these defects.

Nmus Δ*pilU* and Δ*pilT2* can partially retract Tfp; their piliation is normal, and they colonize mice and adhere to mouse cells like Wt. However, they colonize mice in smaller numbers, suggesting that PilU and PilT2 do play a role in colonization, albeit a lesser one. We were surprised by the moderate reduction in colonization frequency of the Δ*pilTU* mutant compared to Δ*pilT*’s significant decrease. It could be possible that PilT2 can compensate *in vivo* for the loss of both PilT and PilU. This requires that PilT2 functions independently from PilT. However, overexpression of PilT2 in an Nme Δ*pilT* background could not rescue Tfp-associated phenotypes (39). An alternate hypothesis is that compensatory upregulation of a secondary adhesin partially compensates/masks the effect of Δ*pilTU*. More work is needed to define the function of PilT2 in these contexts.

Microcolony formation by Nmus Tfp retraction mutants provides clues to colonization phenotypes (Fig. 5D). Compared to Wt, Δ*pilT* forms larger microcolonies with a higher cell density, and these structures detach from the substrate more readily when exposed to fluid flow. The aberrant structure of Δ*pilT* microcolonies is expected as Nme Δ*pilT* and Ngo Δ*pilT* microcolonies are also larger (35, 39, 55), and PilT-dependent Tfp retraction pulls bacteria together within a microcolony, thereby helping to define the size and shape of the structure (48, 55, 56). The fragility of Δ*pilT* microcolony adherence recalls the colonization defect of the mutant (see below).

Conversely*, pilU* has little influence on microcolony formation, based on the parameters examined in this study. Δ*pilU* forms microcolonies like those of Wt, whereas Δ*pilTU* forms microcolonies like those of Δ*pilT*. Surprisingly, Δ*pilT2* forms microcolonies that are larger and denser even than Δ*pilT* microcolonies. Yet, Δ*pilT2* microcolonies withstand fluid flow forces as well as Wt microcolonies.

**Fig 6:**
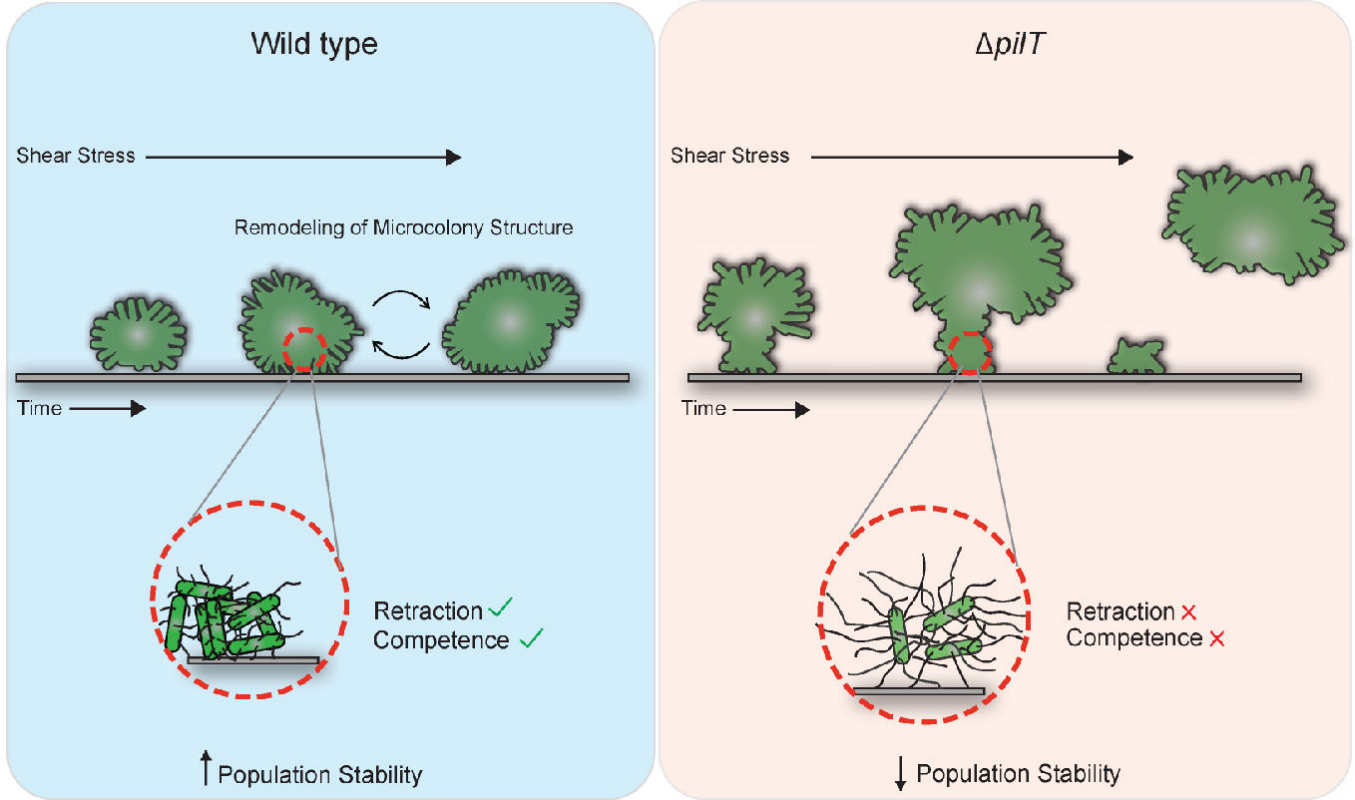
Model depicting mechanisms that may underlie Tfp retraction-dependent colonization defects of *N. musculi*. Tfp retraction enables Nmus to form microcolonies that adhere properly to substrates/mucosal surfaces and to withstand environmental stresses such as force from fluid flow (left column). This resistance to environmental stressors may be due in part to retraction-dependent activation of yet-to-be-defined mechanosensitive transcriptional regulatory networks. Tfp retraction mutants may also form improperly structured microcolonies, increasing the susceptibility of segments of these structures to removal by fluid flow (right column). The remaining adherent cells divide, reforming poorly structured microcolonies that are sensitive to removal by fluid flow, resulting in the cyclic detection phenotype. In Tfp retraction mutants, putative mechanosensitive regulatory networks are not activated, resulting in maladaptation to changing conditions within the microcolony itself and/or inability to respond to external stress imposed by the host microenvironment.

Can we make sense of the various *in vivo* and *in vitro* phenotypes of Nmus Tfp retraction mutants? Based on this and previous studies, we propose a model to explain how Tfp retraction influences Nmus colonization (Fig. 6). Compared to Wt controls (left panel), immobile Δ*pilT* cells cannot form normal microcolonies (right panel). Unable to retract Tfp, replicating cells passively cluster into static, poorly anchored aggregates. The colonization and cell adherence defect of Δ*pilT*, the decreased Δ*pilT* burdens in the few mice harboring the mutant, and the susceptibility of Δ*pilT* microcolonies to removal by fluid flow force stem from the formation of mutant “pseudo-microcolonies” that cannot function properly. In Δ*pilU*, Δ*pilT2*, and *pilT_L201C_*, even a partial ability to retract Tfp enables them to colonize and persist in mice in small numbers.

This model also invokes the idea of Tfp as a pseudo-sensory organ, based on observations of the *P. aeruginosa pil-chp* system (57–59). There, interaction of retracting Tfp with a surface generates mechanical forces that are transmitted along the pilus to the bacterial cell, triggering a signaling cascade that regulates virulence gene expression (58). Although *Neisseria* do not have *pil-chp* orthologs, there is evidence that Tfp retraction is integrated into a wider regulon. Several Ngo genes are differentially expressed in Δ*pilT* (60). The Nme Tfp fiber is modified upon bacterial contact with host cells, suggesting that active substrate sensing by bacteria alters Tfp function (61). It is tempting to speculate that in Nmus retraction mutants a Tfp-dependent regulatory network is interrupted and bacterial adaptation to the host environment impeded. Finally, the activation of mechanosensitive host-cell pathways by Tfp retraction may also contribute to the microenvironment encountered by Nmus on the mucosa. More work is needed to investigate whether commensal *Neisseria* trigger these pathways *in vivo*.

Our model is compatible with the Δ*pilT* and *pilT_L201C_* cyclic detection phenotype and the inconsistency of CV retention. In the former case, shear force causes detachment of segments of mutant microcolonies from the mouse epithelium, dramatically reducing the number of cell-associated bacteria. The remaining adhered cells replicate, rebuilding the microcolonies, which increase recoverable CFUs at later time points. In the latter case, variable CV staining is caused by mutant microcolonies detaching from the microtiter plate during sample processing.

Tfp is likely the first point of contact between *Neisseria* and their cognate host. *Neisseria* Tfp-associated host proteins identified to date include CD46, CD147, and β2-adrenergic receptor. Tfp is speculated to also bind non-specifically to host cell surfaces through surface charges (62–66). Many non-Tfp adhesin/receptor pairs are known to promote Ngo- and Nme-host interactions, *s.a*. Opa-CEACAMs, NhhA-heparin sulfate, and NadA-β-1 integrin (67). We have identified putative Ngo and Nme adhesin orthologs in Nmus [Table S2 and reference (68)]. It is therefore likely that non-Tfp adhesin(s) also contribute to Nmus colonization of the mouse. The logical extension of our model suggests that PilT-dependent Tfp retraction creates local condition(s) that optimize adhesin/receptor pairing, driving mucosal colonization.

Much work is needed to fully understand the role of Tfp retraction in niche development. Regulatory elements enabling response to physical and environmental cues are unknown. Measuring Tfp retraction forces of different *Neisseria* species and motor mutants may help determine whether/how such forces influence biological outcomes. Identifying the presence of Tfp-related phase variable genes in Nmus, if they exist, may also shed light on the phenotypes we observed *in vivo.* Our work represents a first step toward understanding Tfp function *in vivo* and expands our knowledge of the importance of Tfp retraction in infection biology of commensal and pathogenic *Neisseria*.

## MATERIALS AND METHODS

### Bacterial culture

Unless stated otherwise, *Escherichia coli* were grown in LB medium at 37°C, and *Neisseria* were grown in GC medium with Supplements I and II at 37°C/5% CO_2_ (69). *Neisseria* inocula originate from a single colony on GCB Rif 40 µg/mL and incubated for 48 hours at 37°C/5% CO_2_. Rough morphotype colonies were lawned on GCB Rif agar and incubated for 18 hours at 37°C/5% CO_2_. Lawns were harvested into GC broth, and OD_600_ was measured. Unless noted, cells were diluted with unsupplemented GC broth and grown in GC with Supplements I and II. CFUs were determined after 48 hours incubation.

### Strain construction

Tables S3 and S4 list strains, plasmids, and primers. Strains were constructed using standard cloning techniques. PCR was performed using Phusion HiFi (New England Biolabs) and GoTaq Green (Promega) master mixes. Gel extractions were performed using a GeneJET kit (ThermoFisher). Ligations were performed with T4 ligase (Invitrogen). Gibson assembly was conducted using an NEBuilder Hifi DNA Assembly Cloning kit (NEB) per manufacturer’s instructions. Bacterial transformation and electroporation were conducted as previously described (69).

Construction of Δ*pilT* and its complement is described (43). Δ*pilT2* was constructed with a synthesized geneBlock (IDT) that replaces the coding sequence with a chloramphenicol resistance marker and 300 bp of upstream and downstream flanking sequence. The fragment was cloned into pCR Blunt (Invitrogen) before transformation into Nmus. To construct Δ*pilU*, we amplified its flanking sequences with primers KR71, KR73, KR74, and KR76, and an erythromycin cassette was amplified from pMR68 using KR72 and KR75. The fragments were combined using Gibson assembly. Δ*pilTU* was created similarly; its flanking sequences were amplified with primers KR66, KR67, KR69, and KR70, and the kanamycin cassette was amplified from pMR68 with primers KR65 and KR68. The fragments were assembled using Gibson assembly. Final constructs were amplified from the assembly reaction using the outer flanking primers Δ*pilU,* KR71, and KR76 and Δ*pilTU,* KR67, and KR70 before transformation into Wt Nmus.

To complement Δ*pilT2*, Δ*pilU*, and Δ*pilTU*, PCR and Gibson assembly were used to insert an erythromycin (*pilT2*) or chloramphenicol (*pilU* or *pilTU*) marker into the 3′ UTR of each operon using primers KR61, KR63, KR124, KR125, and KR128-KR133. Constructs were introduced into AP2365 by transformation and selected on appropriate antibiotics. Fragments containing the Wt gene and resistance markers were amplified by PCR using KR67 and KR138 to complement Δ*pilTU*; KR71 and KR138 to complement Δ*pilU*; and KR31 and KR32 to complement Δ*pilT2*. Each fragment was gel-purified and recombined into recipient mutant strains by transformation (Δ*pilT2* and Δ*pilU)* or electroporation (Δ*pilTU)*. Constructs were confirmed by antibiotic selection, PCR, and sequencing.

*pilT*_*L201C*_ was created by synthesizing a 1.2-kb geneBlock (IDT) containing the 3′ end of *pilT* containing a coding sequence that encoded a leucine to cysteine substitution at residue 201, followed by 200 bp of downstream sequence. This fragment was inserted into pGemT-Ez (Promega) containing the 5′ end of *pilT* amplified with primers KR1 and KR2. The kanamycin cassette from pMR68 was amplified with primers KR3 and KR4 and cloned into the AfeI site of the *pilT*_*L201C*_ 3′ region. Linearized plasmid was transformed into Wt Nmus, and Km^R^ clones were selected for confirmation by PCR and sequencing. Isolates from two independent transformations were used in all experiments.

### Growth curves

Overnight lawns were suspended in supplemented GC broth with 5 mM Na_2_CO_3_, adjusted to OD_600_ = 0.125, inoculated into 96-well plates in four technical replicate wells, and incubated in a plate reader at 37°C with shaking for 45 seconds, then OD_600_ was determined hourly.

### Transformation assays

Transformations were conducted as previously reported (43, 69). We incubated 2.0 × 10^6^ CFUs with/without 1 µg of AP2098 DNA in six-well plates for 4 hours. Transformants were diluted and plated on GCB Rif 40 µg/mL and GCB Streptomycin 100 µg/mL, incubated for 48 hours, and CFUs were determined. Transformation frequencies were calculated as (#Strep^R^ CFUs/Total #Rif^R^) CFUs/1 µg DNA.

### RNA extraction and RT-qPCR

Cells from 18-hour lawns at a final OD_600_ = 0.05 were inoculated into 100 mm dishes in supplemented GC broth, incubated for 18 hours, and harvested in RNA-Protect (Invitrogen). Total RNA was extracted using Trizol reagent per the manufacturer’s instructions. DNA was removed using DNA-Free (Ambion). cDNA was synthesized from DNAse-treated RNA using a Superscript II First Strand cDNA Synthesis Kit (Invitrogen). *pilE* mRNA was measured using the primer KR139-KR140 with Sybr Select master mix (Invitrogen) on ABI 7300, using 16S primers MR493 and MR494 as an internal control. Expression was analyzed using ABI SDS software version 1.4 and the Pfaffl ddCT method (70).

### Pilin determination

Pilin was determined from extracellular pili extracts from 20- to 24-hour cultures using 4 × 10^9^ CFUs as previously described (71). Bacteria were suspended in ice-cold 0.5 M ethanolamine in PBS (pH = 10.5), vortexed, and iced. Bacteria were removed by centrifugation at 2,000 × *g* for 15 minutes. Supernates were recentrifuged, and pili were precipitated overnight at 4°C in PBS saturated with ammonium sulfate. Pili were pelleted by centrifugation at 4°C, resuspended in 0.5 M ethanolamine-containing PBS, and boiled in 4× Laemelli buffer before by SDS-PAGE (16%). The gels were Coomassie-stained and imaged on Licor Odyssey CLx. Nmus Δ*pilE* was the negative control.

### Cell adhesion

Adhesion assays are previously described (72). CMT-93 mouse rectal epithelial cells were grown in high-glucose DMEM with L-glutamine and pyruvate (Gibco) with 10% FBS. Bacteria were inoculated in triplicate wells of six-well plates, MOI of 10, and incubated for 4–16 hours. Monolayers were washed 2× with PBS and lysed with 0.05% sterile saponin, and lysates were collected. Supernatant and cell-associated fractions were diluted and plated on GCB plates for CFU. The adhesion index was calculated as (Cell-associated CFU/Total CFU) × 100.

### Biofilm assays

Biofilm development was assayed per published methods (73). A suspension of 1 × 10^6^ CFUs/mL in supplemented GC broth was inoculated into each of five wells of a 96-well plate and incubated for 24 hours. Supernates were discarded, and plates were washed with dH_2_O and incubated with 0.3% CV at RT. Supernates were discarded, and the plates were washed and dried for at least 24 hours at RT. Bound dye was resolubilized in 30% glacial acetic acid, and absorbance at 550 nm was measured on a plate reader.

### Bacterial detachment assays

For each strain, bacterial lawns were adjusted to OD_600_ = 0.0025 in supplemented GC broth and inoculated into an Ibitreat μ-slide VI 0.4 (Ibidi) in duplicate channels. Slides were incubated for 24 hours, then processed. Each channel was attached to a low-flow peristaltic pump and perfused with GC broth at rates of 0.2 and 0.6 dyn/cm^2^ of shear stress per the manufacturer’s specifications. Effluent was collected from each channel for 2 minutes, diluted and plated on GCB Rif 40 µg/mL plates. CFUs were counted after 48 hours of incubation to assess adherence and normalized to CFU/mL of effluent for comparison between flow rates.

### Microcolony and biofilm characterization

We resuspended 18-hour lawns to OD_600_ = 0.0025 in supplemented GC broth, inoculated them into duplicate channels of an Ibitreat μ-slide VI 0.4 (Ibidi), and incubated them for 24 hours. Phase contrast images were captured on a Nikon Eclipse Ti. Image analysis of 20–24 fields/strain was conducted using Nikon Elements Advanced Research software. Binary thresholds were applied to each image, and microcolony area was calculated using the automated area functions. After the 24-hour timepoint, slides were processed for confocal imaging. Microcolonies were fixed with 2% paraformaldehyde in Dulbecco’s PBS, stained with DAPI, and mounted in Ibidi mounting media before imaging on a Zeiss 880 LSM confocal microscope. Z-stack images were captured from a total of 12 representative fields per mutant from three independent experiments. Images were analyzed using BiofilmQ (74). Global biofilm properties and individual microcolony measurements were calculated from preprocessed files with and without segmentation by cube dissection, respectively. Scanning electron micrographs of 20-hour cultures were captured on a Hitachi S-4800 at the Kuiper-Arizona Laboratory for Astromaterials Analysis after processing using previously described methods (75).

### Animal experiments

Six-week-old female CAST/EiJ and A/J mice were from Jackson Laboratory. Mice were screened for endogenous *Neisseria* and acclimated to the facility for 2 weeks, then inoculated as previously described (76). Weekly sampling of the oral cavity and fecal pellets was conducted for 8 or 16 weeks depending on the experiment. CFUs were determined by plating diluted samples on GCB Rif 40 µg/mL. At endpoint, animals were humanely sacrificed, and tissues were snap-frozen in LN_2_ and stored at −80°C.

### DNA extraction and 16S amplicon sequencing

DNA was extracted from the chyme of snap-frozen gastrointestinal tissues using a modified bead-beating protocol (77). Luminal contents of each tissue were collected in sterile Eppendorf tubes and weighed. The chyme was resuspended in equal volumes of lysis buffer (200 mM NaCl, 200 mM Tris-HCl, pH 8.0, 20 mM EDTA, and 4% SDS) and 25:24:1 mixture of phenol, chloroform, and isoamyl alcohol. Sterile 0.1-mm silica disruptor beads were added to each sample. The samples were heated to 95°C and shaken at high speed on a bead-beating platform vortex attachment. DNA was purified from the aqueous phase of each sample by two rounds of phenol-chloroform extraction, RNAse treatment, and ethanol precipitation.

Purified DNA was sent to the W.M. Keck Ecological and Evolutionary Genetics Facility at the Marine Biological Laboratory (Woods Hole, MA) and sequenced using bacterial rRNA primers 518F and 926R spanning the V4-V5 region of the 16S rRNA on an Illumina MiSeq instrument. Initial analysis was conducted via the VAMP’s automated pipeline. Reanalysis was conducted using the QIIME2 suite (78). Briefly, samples were joined, denoised, and assembled using Deblur (79), MAFTT, and FastTree (80, 81). Rarefaction and diversity indices were calculated using the q2-diversity plugin using 6–8k reads (82, 83). Diversity metrics were compared by pairwise Kruskal-Wallis and PERMANOVA (82, 83). Taxonomy was assigned by a Bayes classifier trained on a SILVA 138v 99 reference library using the V4-V5 region (84, 85). Comparisons of population composition were conducted using ANCOM at the OTU level (86).

### Statistics

Statistics were completed for all experiments using GraphPad Prism V9. Normality of samples was assessed by Shapiro-Wilk and D’Agostino tests. Parametric samples were analyzed by ordinary one-way ANOVA and Tukey’s comparison to all samples or Dunnett’s comparison to Wt controls. Non-parametric samples were analyzed by Kruskal-Wallis test with Dunn’s post hoc comparison. Colonization phenotype frequencies were examined by Fisher’s exact test. Bacterial burden variability for colonized Wt vs L201C inoculated mice was examined by two-tailed Welch’s unpaired *t* test on mean standard deviation of per-mouse log-transformed burdens.

## Data Availability

Data are available in the Sequence Read Archive (SRA) under BioProject accession PRJNA986897.

## SUPPLEMENTAL MATERIAL

Figure S1 mbio.02792-23-s0001.pdf Alignment of PilT, PilU, and PilT2 of *N. musculi* and human-adapted *Neisseria* species.

Figure S2 mbio.02792-23-s0002.pdf Analysis of bacterial burdens of *pilT*_L201C_ inoculated mice.

Figure S3 mbio.02792-23-s0003.pdf Analysis of microbiota of *pilT*_L201C_ inoculated mice.

Figure S4 mbio.02792-23-s0004.tif In vitro characterization of *N. musculi* Tfp retraction mutants.

Figure S5 mbio.02792-23-s0005.pdf Tfp mutant microcolony analysis.

TABLE S1 mbio.02792-23-s0006.pdf Tables S1 to S4.

ASM does not own the copyrights to Supplemental Material that may be linked to, or accessed through, an article. The authors have granted ASM a non-exclusive, world-wide license to publish the Supplemental Material files. Please contact the corresponding author directly for reuse.
