# Supplementary material for: Type IV pilus retraction is required for Neisseria musculi colonization and persistence in a natural mouse model of infection
Source: mBio. 2023 Dec 12;15(1):e02792-23. doi: 10.1128/mbio.02792-23 (PMC10790696; doi:10.1128/mbio.02792-23)
Supplement: Figure S1 — Alignment of PilT, PilU, and PilT2 of N. musculi and human-adapted Neisseria species. [file mbio.02792-23-s0001.pdf]

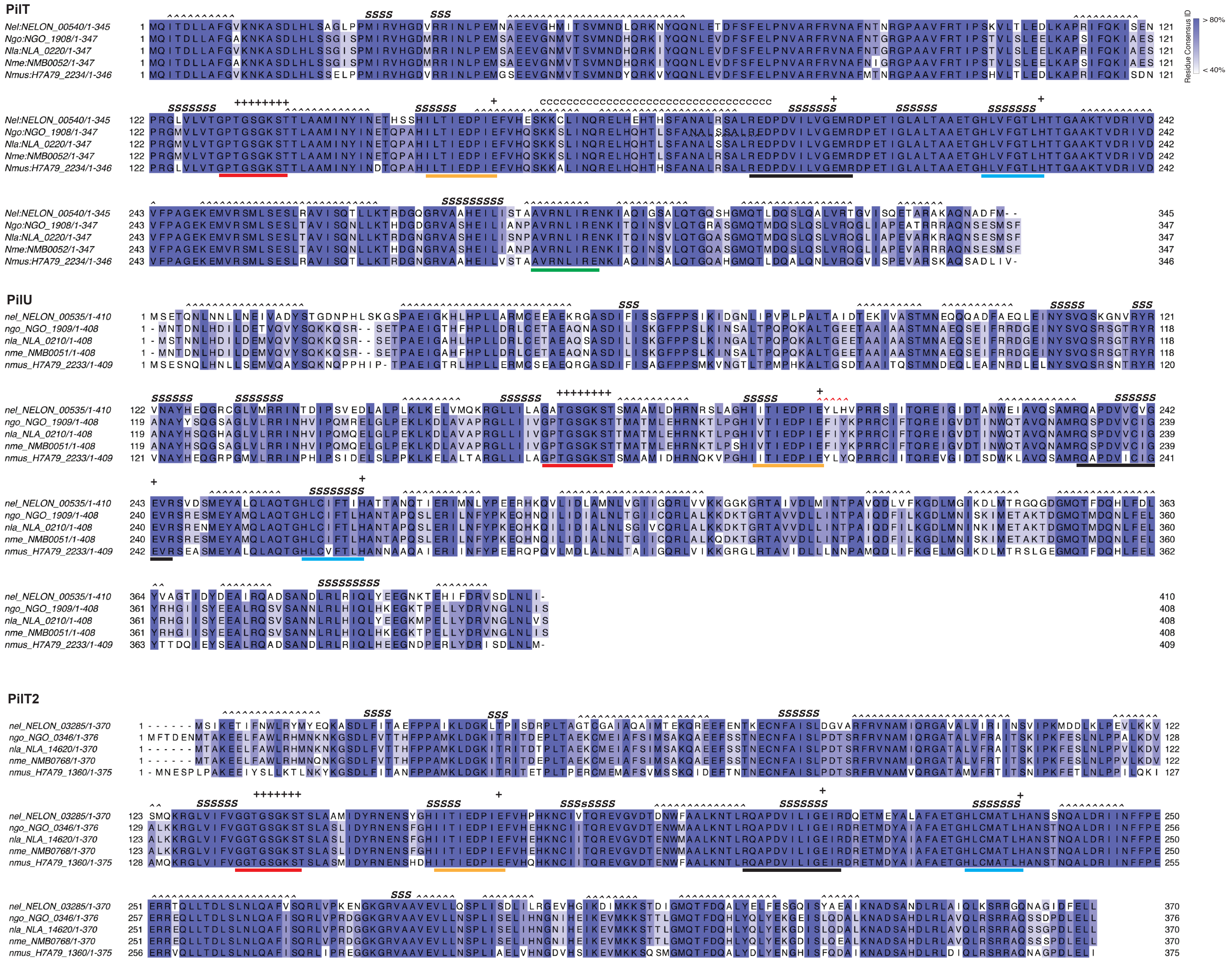

Supplemental Figure 1

PilT, PilU and PilT2 of *N. musculi* and human-adapted *Neisseria* species are highly conserved. Alignment of PilT, PilU and PilT2 amino acid sequences of Nmus and their orthologs in *N. lactamica* 002-06, *N. elongata glycolitica* ATCC 29315, *N. meningitidis* MC58 and *N. gonorrhoeae* FA1090. Pairwise comparison of amino acid sequences was conducted by BLASTP, followed by multiple sequence alignment using ClustalW and Jalview. Percent identity of each residue to the consensus is denoted by color, see scale at top right corner of figure. Walker A and B Boxes are denoted by red and black lines, respectively. The Asp box is underlined in orange, and the His box domain is underlined in blue. The AIRNLIRE domain in PilT is underlined in green. Jpred secondary structure prediction was conducted in Jalview and predictions are annotated as follows: ^, alpha-helix; S, beta-sheet; c, coil-coil. Only predictions with a confidence above 3 are included. Residues implicated in nucleotide binding in crystal structures of PilT in other species are marked with + (23).
