# Supplementary material for: Type IV pilus retraction is required for Neisseria musculi colonization and persistence in a natural mouse model of infection
Source: mBio. 2023 Dec 12;15(1):e02792-23. doi: 10.1128/mbio.02792-23 (PMC10790696; doi:10.1128/mbio.02792-23)
Supplement: Figure S2 — Analysis of bacterial burdens of pilTL201C inoculated mice. [file mbio.02792-23-s0002.pdf]

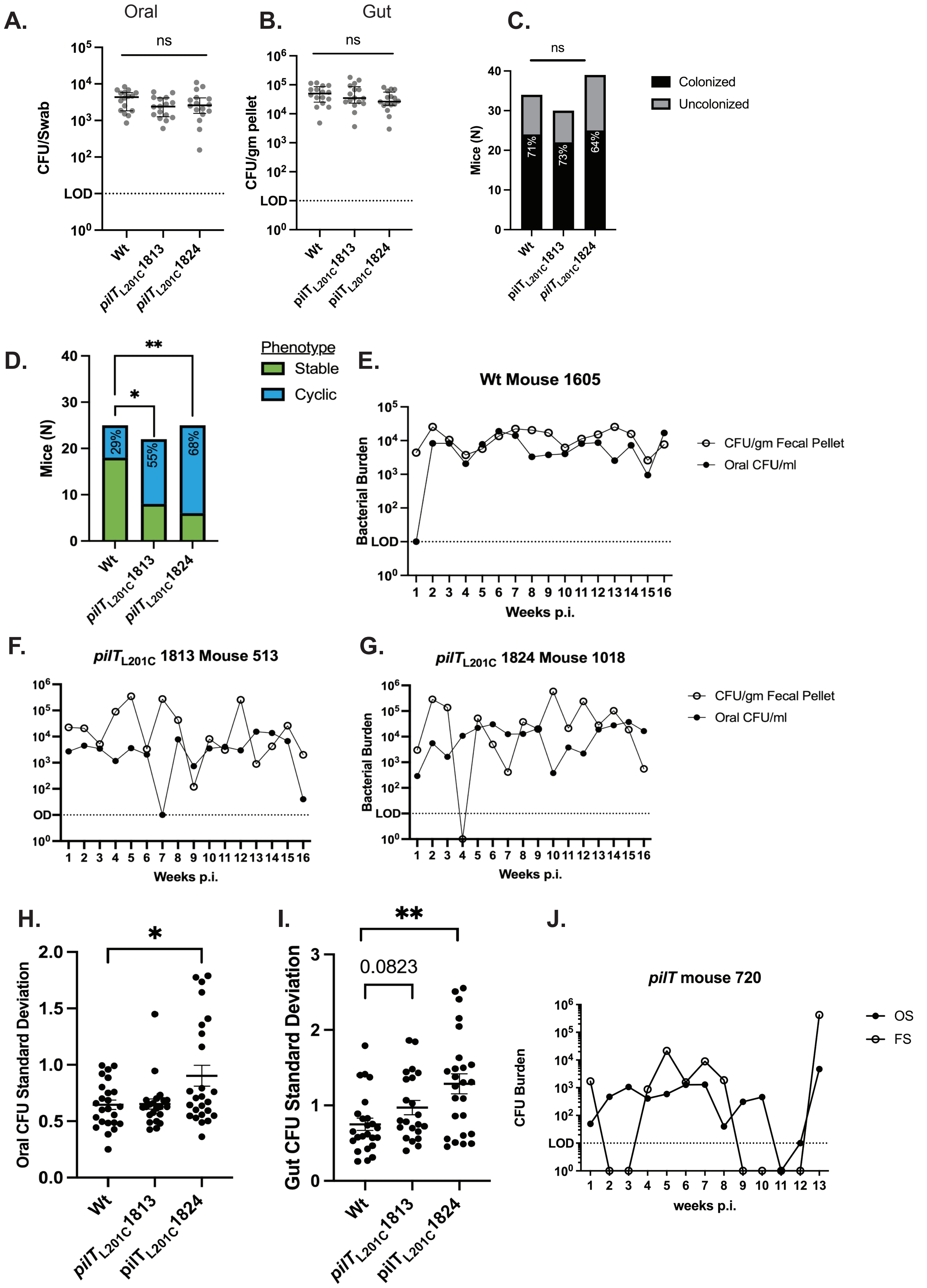

Supplemental Figure 2

A site-directed mutation in the PilT Walker Box B of *N. musculi* results in variability of bacterial burden in CAST/EiJ mice. (A)-(B) Wt Nmus and two independently constructed mutants, $pilT_{L201C}$ 1813 and $pilT_{L201C}$1824, with a leucine to cysteine change in residue 201 within PilT Walker Box B, were inoculated into the oral cavity of CAST/EiJ mice. Oral swabs and fecal pellets were collected for 16 weeks, and samples were plated on selective agar to enumerate CFUs in the oral cavity (A) and gut (B). Dots represent mean weekly CFUs per group, and lines denote the median and interquartile range. Statistical analysis was conducted by Kruskal-Wallis test with Dunn's comparison to Wt. (C) Colonization of CAST/EiJ mice by Wt, $pilT_{L201C}$ 1813, and $pilT_{L201C}$1824. Colonization is defined as mice that are culture-positive for two consecutive time points by 4 weeks post-inoculation. Bars represent colonization frequency, and percentages of colonized animals are noted in the bars. Differences in colonization frequencies were compared by Two-Tailed Fisher's exact test. (D) Persistent colonization phenotypes of Wt, $pilT_{L201C}$1813, and $pilT_{L201C}$1824. Stable colonization phenotype is represented by green bars, and the cyclic detection phenotype by blue bars. Cyclic detection phenotype is defined as increases/decreases in CFU of 1.5 log magnitude, detected for >2 consecutive weeks, after 4 weeks post-inoculation. Statistical comparison was conducted by Two-tailed Fisher's exact test to Wt controls.
(E) Representative stable colonization phenotype of Wt Nmus. Oral CFUs: black circles; fecal pellets, open circles. (F) Representative cyclic detection phenotype of $pilT_{L201C}$1813.(G) Representative cyclic detection phenotype of $pilT_{L201C}$1824. Oral CFUs: black dots; fecal pellets, open circles. (H)-(I) Plots depicting standard deviation of log transformed oral (H) and gut (I) burdens of individual colonized mice over the 16 week study. Statistical analysis was performed by Unpaired Two-Tailed Welch's t Test comparison to Wt controls.
(J) Representative cyclic detection phenotype of Δ*pilT.* Dots represent CFUs from oral swabs (OS, black circles) and fecal samples (FS, open circles). $^{*}p < 0.05$, $^{**}p < 0.01$. LOD; limit of detection, N=30-39 mice per group. O.S., oral swab, F.S., fecal sample.
