# Supplementary material for: Type IV pilus retraction is required for Neisseria musculi colonization and persistence in a natural mouse model of infection
Source: mBio. 2023 Dec 12;15(1):e02792-23. doi: 10.1128/mbio.02792-23 (PMC10790696; doi:10.1128/mbio.02792-23)
Supplement: Figure S3 — Analysis of microbiota of pilTL201C inoculated mice. [file mbio.02792-23-s0003.pdf]

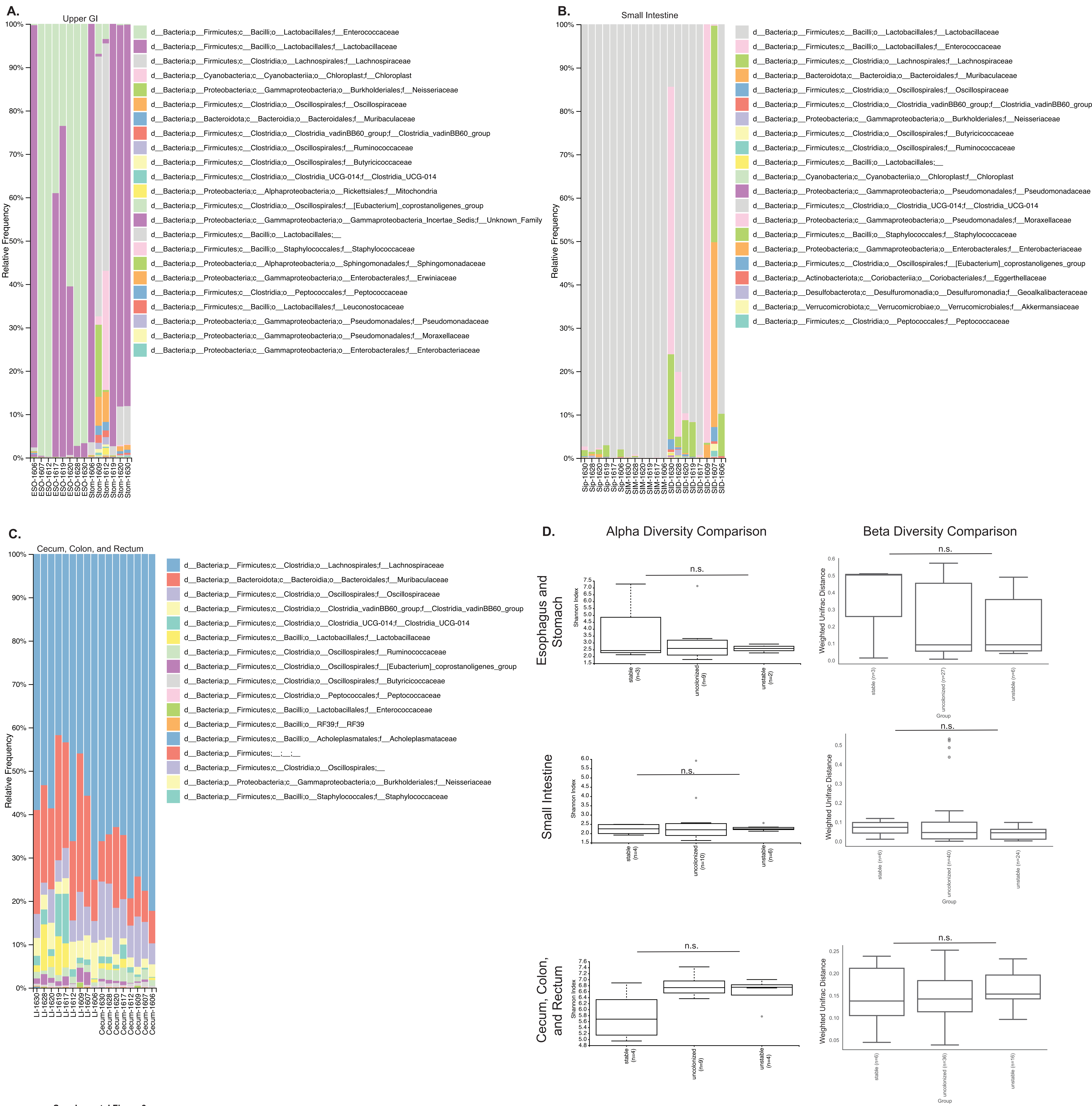

Supplemental Figure 3

The cyclic detection phenotype of *pilT*$_{L201C}$ is not associated with microbiota composition. (A-C) Family level composition of samples grouped by body site. Bars depict relative frequency of features within each sample. Taxonomic classification is defined in the legend for each group. ESO, esophagus; Stom, stomach; SIP, proximal small intestine; SIM, medial small intestine; SID, distal small intestine; LI, colon and rectum. (D) Comparison of alpha and beta diversity metrics between mice displaying stable, cyclic, and uncolonized *N. musculi* phenotypes. Left panel, boxplot depicts median and interquartile ranges of Shannon alpha diversity index within stable, cyclic (unstable), and uncolonized samples. Outliers are plotted as points. Not significant, Kruskal-Wallis pairwise comparison between groups. Right panel, boxplot depicts median and interquartile range of weighted UniFrac distances within stable, cyclic (unstable) and uncolonized samples. Outliers are plotted as points. (N) number of comparisons performed to stable samples in each group. Not significant, PERMANOVA analysis.
