# Supplementary figures and images for: Type IV pilus retraction is required for Neisseria musculi colonization and persistence in a natural mouse model of infection
Source: mBio. 2023 Dec 12;15(1):e02792-23. doi: 10.1128/mbio.02792-23 (PMC10790696; doi:10.1128/mbio.02792-23)

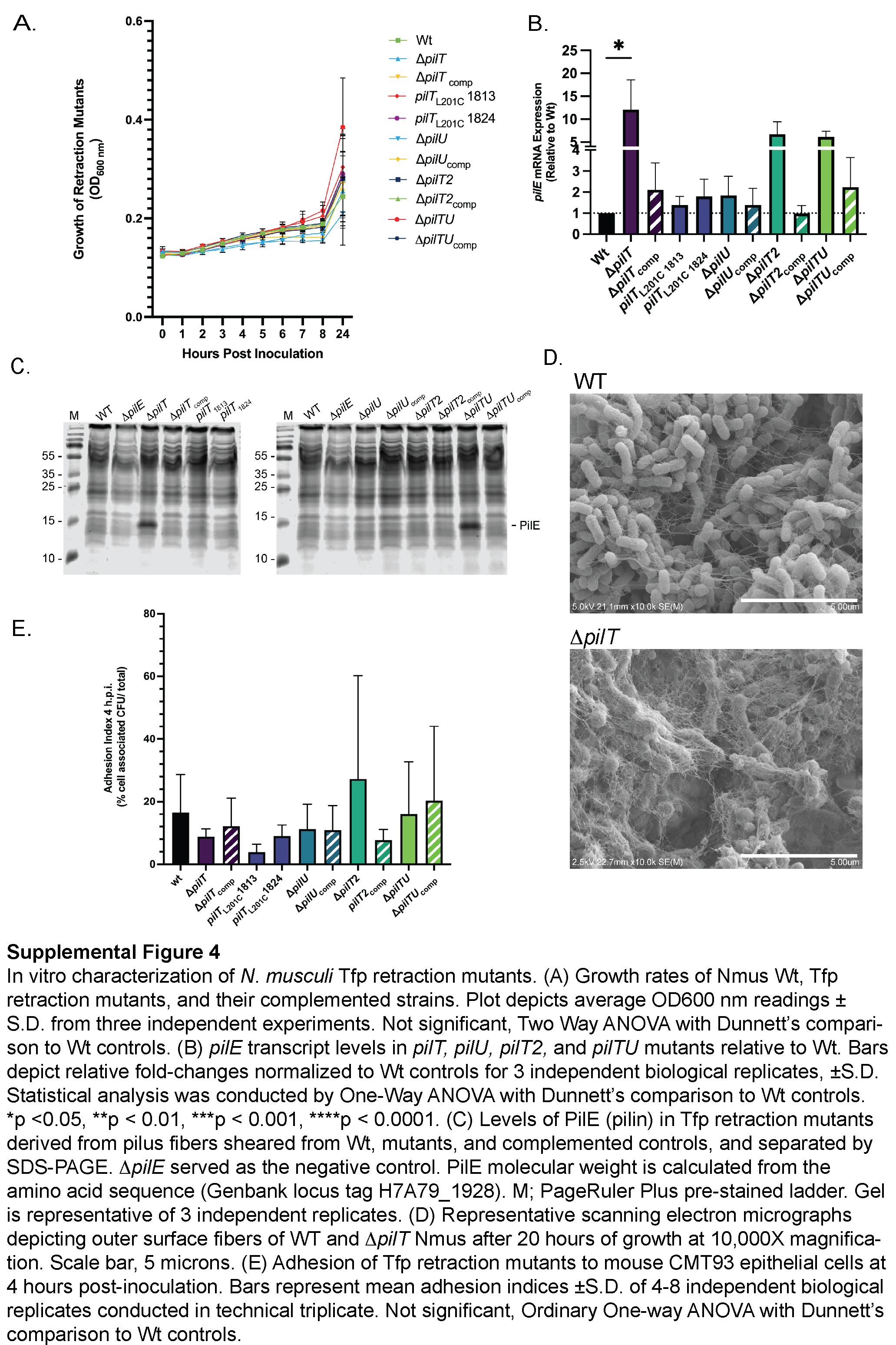

Supplement: Figure S4 — In vitro characterization of N. musculi Tfp retraction mutants. [file mbio.02792-23-s0004.tif]
