# Supplementary material for: Type IV pilus retraction is required for Neisseria musculi colonization and persistence in a natural mouse model of infection
Source: mBio. 2023 Dec 12;15(1):e02792-23. doi: 10.1128/mbio.02792-23 (PMC10790696; doi:10.1128/mbio.02792-23)
Supplement: Figure S5 — Tfp mutant microcolony analysis. [file mbio.02792-23-s0005.pdf]

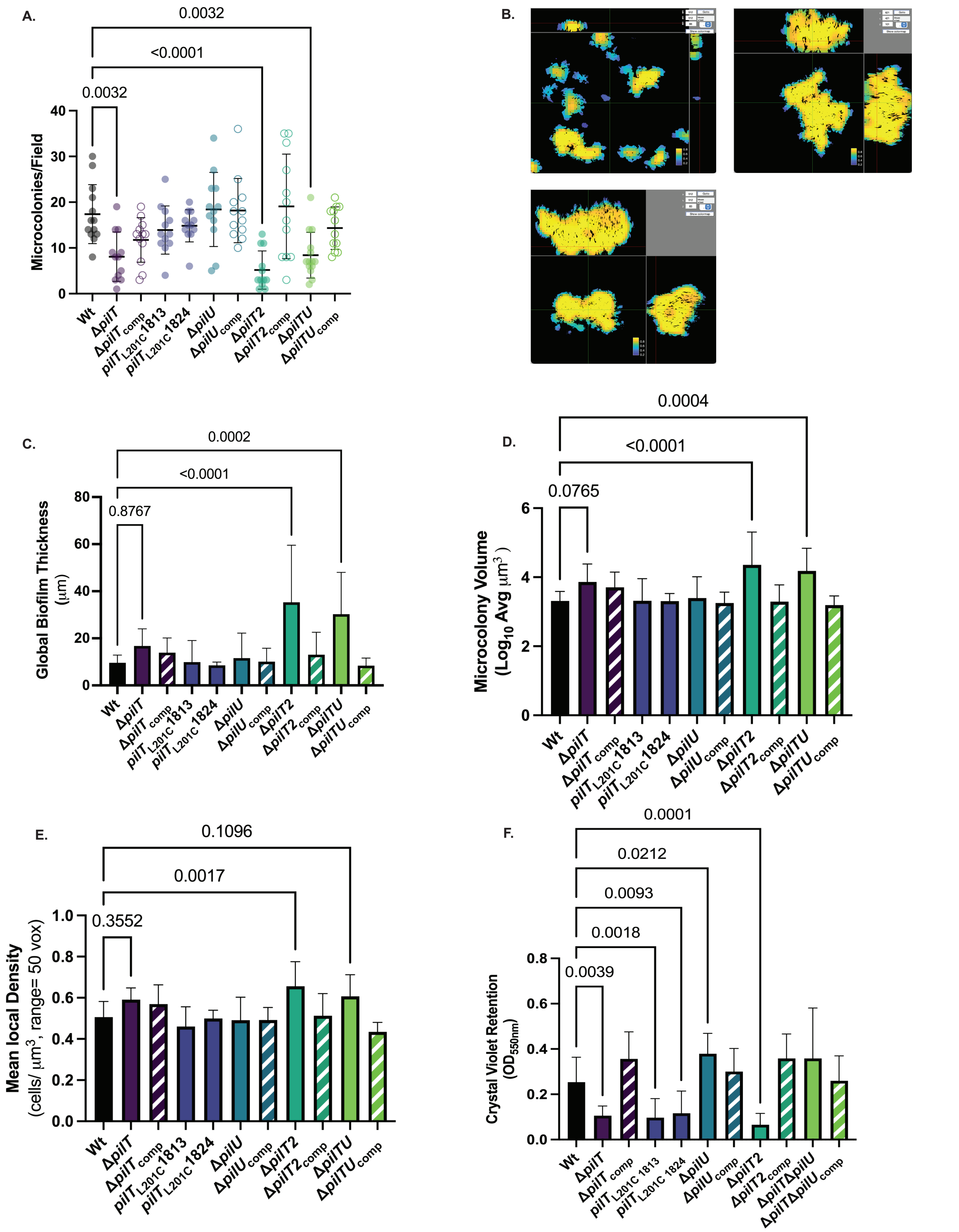

Supplemental Figure 5.
PilT, PilT2 and PilTU *N. musculi* mutants display distinct microcolony phenotypes. (A) Average number of microcolonies visible per field. Dots indicate number of microcolonies identified during BiofilmQ fluorescence thresholding, with a line at the mean ±S.D. (B) Representative orthographs of Z-stacks captured from the samples in (A) after processing for imaging on a Zeiss LSM 880, analyzed by BiofilmQ. Color scale indicates level of local cell density (cells/µm3, in a 50-voxel sphere). (C) Quantification of global biofilm thickness in Nmus Wt, retraction motor mutants, and complemented controls. Bars depict average microcolony volume from 12-14 representative fields per strain, ±S.D. (D) Quantification of the volume of microcolonies formed by Nmus Wt, Tfp retraction mutants and complemented controls. Bars depict average microcolony volume from 12-14 representative fields per strain, ±S.D. (E) Local cell density in microcolonies formed by Wt, Tfp retraction mutants, and complemented controls. Bars depict average local cell density ±S.D., calculated from 12-14 representative fields. (F) Crystal violet retention of Nmus Wt, Tfp retraction mutants, and complemented strains, 24 hours post-inoculation. Bars represent average OD550 nm absorbance of three biological replicates ±S.D. Statistical analysis in (A) and (C)-(F), was conducted by One-Way ANOVA with Dunnett's multiple comparison to Wt controls, and exact p values are annotated on each plot.
