# Supplementary material for: Type IV pilus retraction is required for Neisseria musculi colonization and persistence in a natural mouse model of infection
Source: mBio. 2023 Dec 12;15(1):e02792-23. doi: 10.1128/mbio.02792-23 (PMC10790696; doi:10.1128/mbio.02792-23)
Supplement: TABLE S1 — Tables S1 to S4. [file mbio.02792-23-s0006.pdf]

Supplementary Table 1. *N. musculi* (Nmus) Tfp retraction mutant phenotypes compared to those of analogous mutants in *N. gonorrhoeae* and *N. meningitidis.*

Mutant strains	Piliation	M.C. formation/	Pilin/ *pilE* expression	Retraction	Cell Adhesion	Surface Detachment
Δ*pilT*	=, =	=*, = *	=, =	=, =	↓, ↓	↓, n.d.
pilT$_{L201C}$	n.d, =	n.d., =	n.d., =	n.d., =	n.d., ↓	n.d., n.d.
Δ*pilU*	=, =	=, =*	=, =	=, =	↓,↓	n.d., n.d.
Δ*pilT2*	=, =	=*, n.d.	=, n.d.	=, =	=, n.d.	n.d., n.d.

Phenotypes for *N. gonorrhoeae* are in blue, for *N. meningitidis* are in green. Symbols: ↑, Nmus phenotype is increased; ↓, Nmus phenotype is decreased; =, Nmus phenotype is similar; *, phenotype described qualitatively in *N. meningitidis* or *N. gonorrhoeae*; n.d., not tested in *N. gonorrhoeae* or *N. meningitidis*. Data for *N. gonorrhoeae* and *N. meningitidis* phenotypes are compiled from previous studies(1-4)

Supplementary Table 2: Putative *Neisseria musculi* adhesins

**Nmus Locus**	**Genbank Annotation**	**Ref Locus**	**Gene name**	**% ID: query coverage (species)**
H7A79_0052	Hypothetical protein	NMB2095	Adhesin complex protein ACP	54.4:96 (*N. meningitidis* MC58*)*
H7a79_0089	copC domain protein	TUM12370_06830	*siiEA*	29.9:64 (*S. enterica subs. enterica Choleraesuis)*
H7A79_0198	Hemagglutinin repeat family protein	NMB0492	*hrpA*	45:79 (*N. meningitidis* MC58*)*
H7A79_0208	filamentous hemagglutinin family N-terminal domain protein	NMB0492	*hrpA*	38.7:79 (*N. meningitidis* MC58*)*
H7A79_0209	Hemolysin secretion/activation ShlB/FhaC/HecB family protein	NMB1762	*hrpB*	59:95 (*N. meningitidis* MC58*)*
H7A79_0412	*Neisseria meningitidis* TspB family protein	NMB1548	*tspB*	30:98(*N. meningitidis* MC58*)*
H7A79_1464	fimV N-terminal domain	NMB0341	*tspA*	38.6:50 (*N. meningitidis* MC58*)*
H7A79_1861	hep_Hag family protein	G4D67_18450	*yapN*	32.4:90 (*Y. pestis*)
H7A79_2020	hemagglutinin family protein	FE440_13265	Trimeric autotransporter adhesin Ata	33.4:83(*A. baumannii)*
H7A79_2426	Gram negative porin family protein	NMB2039	*porB**	48.4:100 (*N. meningitidis* MC58*)*

Putative adhesins were collated by BlastP and Uniprot searches of adhesins and adhesin domains (5-7) against the *N. musculi* genome. *, Adhesion associated.

Supplementary Table 3. Strains and plasmids used in this study

Strains and Plasmids	Notes	Source
Ap2365	Rif^R derivative of Ap2031 *N. musculi* type strain.	(8, 9)
Δ*pilT*	Ap2365 Background, Genbank Locus H7A79_2234	(8, 9)
Δ*pilT*$_{comp}$	Ap2365 Background	(8, 9)
Δ*pilE*	Ap2365 Background, Genbank Locus H7A79_1928	(8)
pilT$_{L201C}$1813	Ap2365 Background, Km^R, Rif^R	This Study
pilT$_{L201C}$1824	Ap2365 Background, Km^R, Rif^R	This Study
Δ*pilU*	Ap2365 Background, Erm^R, Rif^R, Genbank Locus H7A79_2233	This Study
Δ*pilU*$_{comp}$	Ap2365 Background, Cm^R, Rif^R	This Study
Δ*pilT2*	Ap2365 Background, Cm^R, Rif^R, Genbank Locus H7A79_1360	This Study
Δ*pilT2*$_{comp}$	Ap2365 Background, Erm^R, Rif^R	This Study
Δ*pilTU*	Ap2365 Background, Km^R, Rif^R	This Study
Δ*pilTU*$_{comp}$	Ap2365 Background, Cm^R, Rif^R	This Study
pMR68		(10)
pCRBlunt:Δ*pilT2 Cm*		This Study
pGEMT: *pilT*$_{L201C}$*Km*		This Study

Supplementary Table 4: Cloning, Sequencing and qRT primers

Primer	Sequence 5'-3'	Use	Source
KR1	GCAGCGAAGCCAGCATG	*pilT*$_{L201C}$	This Study
KR2	CTCGAGGTATGGGCGGTTCT	*pilT*$_{L201C}$	This Study
KR3	TATAAGCGCTATGGACAGCAAGCGAA	*pilT*$_{L201C}$ Afe1	This Study
KR4	ATTAAGCGCTGTCGGTCATTTCGAACC	*pilT*$_{L201C}$, Afe1	This Study
KR65	ACTGAAAACGCAAGGCTACACATT CAGCAAGCGAACCGGAATTGCCA	Δ*pilTU* Km fragment	This Study
KR66	TGGCAATTCCGGTTCGCTTGCTGAAT GTGTAGCCTTGCGTTTTCAGT	Δ*pilTU* 5’ flank	This Study
KR67	CTCGCCGGCCACACACATCAAGCCGCG	Δ*pilTU* 5’ flank	This Study
KR68	AAGGGTGCCGGCCTGTGGTCGG TCATTTCGAACCCCAGAG	Δ*pilTU* Km fragment	This Study
KR69	CTCTGGGGTTCGAAATGACCGA CCACAGGCCGGCACCCTT	Δ*pilTU* 3’ flank	This Study
KR70	GCAGCCGGGAACGGTAGGATGTGGTATGGGCG	Δ*pilTU* 3’ flank	This Study
KR71	GGCCGCGTGGCGGCGCATGAAATTCTGGTGTCG	Δ*pilU 5’* flank	This Study
KR72	CGAAAAATCTCAGGGAAAACACCTGGCGTAA TCATGGTCATAGCTGTTCGATAA	Δ*pilU* Erm fragment	This Study
KR73	TTATCGAACAGCTATGACCATGATTACGCCAGG TGTTTTCCCTGAGATTTTTCG	Δ*pilU 5’* flank	This Study
KR74	CTTGTTTTTCGTGTACCCACAGGCCGGCACCCTTGCCG	Δ*pilU* 3’ flank	This Study
KR75	CGGCAAGGGTGCCGGCCTGTGGGTACACGAAAAACAAG	Δ*pilU* Erm fragment	This Study
KR76	GTAGGATGTGGTATGGGCGGTTCTGCGGCTTAACCGGC	Δ*pilU* 3’ flank	This Study
KR61	TGCCGCATCCGGAGCGCTGTAAGAGGTTCCAACTTTC	*pilTU* comp flank, Afe1	This Study
KR63	GAAAGTTGGAACCTCTTACAGCGCTCCGGATGCGGCA	*pilTU* comp Cm fragment, Afe1	This Study
KR124	TGGCAGGGCGGGGCGTAAAGCGCTAGAGCTTCT	*pilTU* comp flank, Afe1	This Study
KR125	AGAAGCTCTAGCGCTTTACGCCCCGCCCTGCCA	*pilTU* comp Cm, Afe1	This Study
KR138	CAGTCAAAACCTTCCTGCCC	*pilTU* comp outer primer	This Study
KR128	CCTTAACTTGTTTTTCGTGT ACCTTTGTTCTTTTCAGACGGCCTC	*pilT2* comp 3’ flank	This Study
KR129	GAGGCCGTCTGAAAAGAACA AAGGTACACGAAAAACAAGTTAAGG	*pilT2 comp* Erm fragment	This Study

KR130	GCCGTCTGAAAAAACCGCTGGCG TAATCATGGTCATAGC	*pilT2 comp* 5' Flank	This Study
KR131	GCTATGACCATGATTACGCCA GCGGTTTTTTCAGACGGC	*pilT2comp* Erm fragment	This Study
KR132	GCAGCTGCTCACCGACCTTTCACTCAACC	*pilT2 comp 5'* flank	This Study
KR133	CTGCGTTCCAGATTTTCTAAAGTTTCTACAGTTA	*pilT2* comp 3' flank	This Study
KR31	TGGGAAATCGGAAATGAAAA	*pilT2* comp outer primer	This Study
KR32	TTCAACAGCTCGATTTCCTG	*pilT2* comp outer primer	This Study
KR139	ACTTTAACTTGGACACGTAGCA	*pilE RT-qPCR*	This Study
KR140	AAGAGTAGCAGTAGCAGAAGT	*pilE RT-qPCR*	This Study
MR493	ATCCTGGCTCAGATTGAACG	16S	This Study
MR494	CCGCTTTCCTTCTCAAAGTG	16S	This Study
518F	CCAGCAGCYGCGGTAAN		(11)
926R	CCGTCAATTCNTTTRAGT CCGTCAATTTCTTTGAGT CCGTCTATTCCTTTGANT		(11)

References

1. Brown DR, Helaine S, Carbonnelle E, Pelicic V. 2010. Systematic functional analysis reveals that a set of seven genes is involved in fine-tuning of the multiple functions mediated by type IV pili in *Neisseria meningitidis*. Infect Immun 78:3053-63.
2. Hockenberry AM, Hutchens DM, Agellon A, So M. 2016. Attenuation of the Type IV Pilus Retraction Motor Influences Neisseria gonorrhoeae Social and Infection Behavior. MBio 7.
3. Park HS, Wolfgang M, Koomey M. 2002. Modification of type IV pilus-associated epithelial cell adherence and multicellular behavior by the PilU protein of Neisseria gonorrhoeae. Infect Immun 70:3891-903.
4. Wolfgang M, Lauer P, Park HS, Brossay L, Hebert J, Koomey M. 1998. PilT mutations lead to simultaneous defects in competence for natural transformation and twitching motility in piliated *Neisseria gonorrhoeae*. Mol Microbiol 29:321-30.
5. Hung MC, Christodoulides M. 2013. The biology of Neisseria adhesins. Biology (Basel) 2:1054-109.
6. Rhodes KA, Ma MC, Rendon MA, So M. 2022. Neisseria genes required for persistence identified via in vivo screening of a transposon mutant library. PLoS Pathog 18:e1010497.
7. Monzon V, Lafita A, Bateman A. 2021. Discovery of fibrillar adhesins across bacterial species. BMC Genomics 22:550.
8. Ma M, Powell DA, Weyand NJ, Rhodes KA, Rendon MA, Frelinger JA, So M. 2018. A Natural Mouse Model for Neisseria Colonization. Infect Immun 86.
9. Weyand NJ, Ma M, Phifer-Rixey M, Taku NA, Rendón MA, Hockenberry AM, Kim WJ, Agellon AB, Biais N, Suzuki TA, Goodyer-Sait L, Harrison OB, Bratcher HB, Nachman MW, Maiden MC, So M. 2016. Isolation and characterization of *Neisseria musculi* sp. nov., from the wild house mouse. Int J Syst Evol Microbiol 66:3585-3593.
10. Ramsey ME, Hackett KT, Kotha C, Dillard JP. 2012. New complementation constructs for inducible and constitutive gene expression in Neisseria gonorrhoeae and Neisseria meningitidis. Appl Environ Microbiol 78:3068-78.
11. Nelson MC, Morrison HG, Benjamino J, Grim SL, Graf J. 2014. Analysis, optimization and verification of Illumina-generated 16S rRNA gene amplicon surveys. PLoS One 9:e94249.
